# Prediction of the Impaction of Proximal Ureteral Stones: A Critical Evaluation of the Patient- and Stone-Related Factors Affecting the Ureteral Wall Thickness

**DOI:** 10.3390/jcm14176081

**Published:** 2025-08-28

**Authors:** Rasim Guzel, Salih Yildirim, Hikmet Taha Temizkan, Alper Asik, Hikmet Yasar, Kemal Sarica

**Affiliations:** 1 Urology Clinic, Kavacık Medistate Hospital, 34805 Istanbul, Turkey; rasimguzel@hotmail.com; 2 Department of Urology, Sancaktepe Sehit Ilhan Varank Research and Training Hospital, 34785 Istanbul, Turkey; tahatemizkan@gmail.com (H.T.T.); alperasik001@hotmail.com (A.A.); hikmetyasarkdh@hotmail.com (H.Y.); saricakemal@gmail.com (K.S.); 3 Department of Urology, Medical School, Biruni University, 34015 Istanbul, Turkey

**Keywords:** ureteral wall thickness, impacted stone, hydronephrosis, ureteroscopic lithotripsy, predictive factors

## Abstract

**Aim:** The aim of this study was to evaluate the patient-, stone-, and anatomy-related factors that may influence the impaction status of proximal ureteral stones, with a particular focus on the ureteral wall thickness (UWT) as a radiologic surrogate marker of chronic irritation. **Materials and Methods:** A total of 115 adult patients who underwent ureteroscopic treatment for impacted proximal ureteral stones between January 2021 and December 2023 were retrospectively analyzed. The demographic data, comorbidities, stone characteristics (volume, location, and HU value), and anatomical parameters (hydronephrosis grade, proximal ureteral diameter, and UWT) were evaluated using non-contrast computed tomography (NCCT). The correlations between the UWT and both patient- and stone-related variables were assessed using linear and logistic regression analyses. **Results:** The mean patient age was 45.3 ± 13.8 years, with a male-to-female ratio of 2.11. A significant positive correlation was observed between the UWT and hydronephrosis grade (*p* = 0.002), presence of comorbidities such as hypertension or diabetes mellitus (*p* = 0.005), and stone volume (*p* = 0.005). A larger stone diameter and length were also significantly associated with increased UWT (*p* = 0.014 and *p* = 0.005, respectively). However, no statistically significant correlation was found between the UWT and stone density (*p* = 0.614) or the duration of stone presence (*p* = 0.987). **Conclusions:** Increased ureteral wall thickness appears to be a strong indicator of stone impaction severity and is positively associated with hydronephrosis, comorbid conditions, and stone size. These findings support the potential clinical utility of UWT in preoperative planning and treatment selection for impacted upper ureteral stones. Further prospective studies are warranted to validate these observations and explore their implications for procedural success and complication risk.

## 1. Introduction

Impacted upper ureteral stones causing varying degrees of obstruction differ from other calculi located in the ureter, not only regarding their clinical symptoms but also their management and follow-up. Impacted ureteral stones are commonly defined as stones that remain lodged in the same position within the ureter for an extended period—typically more than one to two weeks—resulting in local inflammation, edema, and potential changes in the ureteral wall structure, such as increased thickness or fibrosis [1]. It has been shown that by remaining in the same position for a certain period of time, these stones can irritate and cause inflammatory reactions in the surrounding ureteric wall. Reactions in the inflammatory phase can cause edematous changes, hypertrophy, and fibrotic alterations over time and be responsible for the increased ureteral wall thickness [1].

The reported literature data have demonstrated well that chronic irritation, mainly caused by the proliferation and infiltration of inflammatory cells, will change the anatomical characteristics of the ureteral wall around the impacted stone, a process that will end with the thickening of the wall. Without timely intervention, these alterations and the subsequent formation of stricture may lead to complications such as hydronephrosis [2]. Among ureteral calculi, proximal stones present unique clinical challenges. They are more prone to becoming impacted, less responsive to shock wave lithotripsy (SWL), and often necessitate technically demanding ureteroscopic interventions [1].

The anatomic natural ureteral wall thickness is around 1 mm [3], and it tends to increase as a result of impaction (embedment), which causes edema, inflammation, and hypertrophy, leading to polyp formation in this portion of the involved ureter. Based on this fact, a number of studies have evaluated the possible effect of stone impaction on the UWT value assessed around the stone in the ureter [4,5,6].

In light of the well-documented changes in the ureteral wall surrounding the embedded stone, the resultant increased ureteral wall thickness (UWT) has been accepted as a noninvasive form of assessment and grading of stone impaction. The data published in the literature have clearly shown that the changes in the ureteric wall, as demonstrated by the assessment of UWT where the stones are present, are considered an important factor affecting the spontaneous passage rates of ureteric calculi and the course/outcomes (complications) of both SWL as well as ureteroscopic stone management. Finally, in terms of the tissue edema and ischemia in the ureteral mucosa induced by stone irritation, UWT was also determined to be helpful in the prediction of double-J stent insertion alongside the impacted stone(s) [7,8,9].

However, despite the detailed evaluations that indicate the clinical value of UWT as a reliable noninvasive predictor, there is no relevant literature focused on the role certain body- and impacted stone-related parameters play in the formation and extent of the ureteral wall thickening.

As demonstrated in prior studies [7,9], the UWT has been recognized as a valuable predictor of stone impaction and procedural outcomes. Our study expands on these findings by simultaneously analyzing a comprehensive set of both patient- and stone-related factors contributing to UWT.

In this study, we aimed to evaluate certain calculi-, upper tract anatomy-, and body-related factors that may affect the impaction status of obstructing upper ureteric stones, mainly focused on the (UWT) values assessed at the stone site.

## 2. Patients and Methods

In total, 115 adult cases who were treated for the removal of impacted proximal ureteral calculi in our department were evaluated. The data obtained from the departmental files were evaluated in a retrospective manner from July 2023 to December 2024, following the ethical approval granted on 14 June 2023.

After obtaining a detailed history, particularly regarding the presence of stone disease, and conducting a thorough urogenital examination, biochemical tests, along with urinalysis and a culture antibiogram test, were carried out and documented in all cases. Prior to all URS procedures, non-contrast computed tomography (NCCT) was performed to assess the patient’s body- and ureteral calculi-related parameters, including the size, location, position, and hardness (HU value). Additionally, the UWT value at the stone site, ureter diameter in the upper (proximal) part of the stone (PUD), and the degree of hydronephrosis were also assessed. Lastly, the position (location status: horizontal or longitudinal) of the stones in the ureter was also carefully evaluated on the CT images in all cases. The presence and degree of hydronephrosis were evaluated on the NCCT sections of all cases assessed, and the degree of hydronephrosis was classified according to the Onen system for grading (grade 0–4) [1]. The ureteral wall thickness (UWT) was measured manually at a single point, representing the maximal wall thickening adjacent to the stone, using axial non-contrast CT images. All measurements were performed by a single experienced radiologist who was blinded to the clinical and outcome data. No specific software was used; measurements were conducted using the standard PACS tools available in our radiology system. The stone position was assessed on axial NCCT images. The stones were categorized as vertical if their long axis was parallel to the ureteral lumen and horizontal if perpendicular. This classification aimed to evaluate whether the orientation of the stone may influence the degree of ureteral wall contact and thereby affect the impaction severity.

The duration of stone presence was determined retrospectively based on clinical anamnesis, using the time interval between patient-reported symptom onset (e.g., flank pain) and the date of CT imaging, as documented in hospital records.

The inclusion criteria were as follows: age ≥ 18 years, presence of impacted proximal ureteral stone confirmed on NCCT, and availability of complete preoperative imaging and clinical data. Exclusion criteria included age < 18 years, pregnancy, congenital or acquired urinary tract anomalies, and incomplete records or missing imaging. All patients underwent surgery within 7 days (mean 3.2 ± 1.4 days) following CT imaging.

The possible correlations between the above-mentioned patient-, radiology-, and anatomy-related factors and the value of the UWT have been well evaluated.

**Figure 1:**
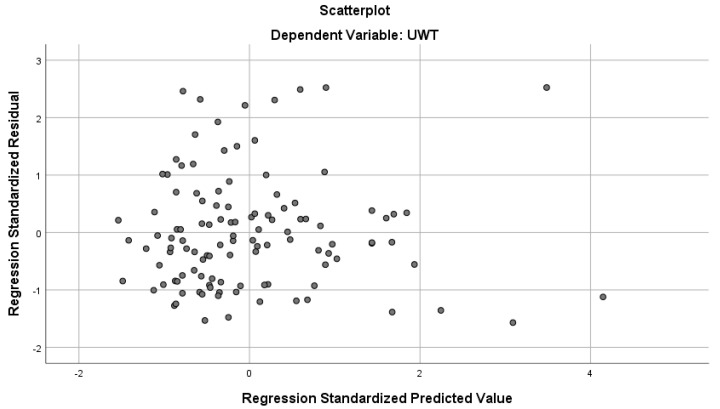
Scatterplot of standardized residuals versus standardized predicted values demonstrating no major pattern indicating heteroscedasticity.

IBM SPSS version 26 software was utilized for statistical analysis. Descriptive statistics were used to describe the demographic characteristics. Measures of central tendency and dispersion (mean and standard deviation) were calculated where appropriate. Student’s *t*-test was used for preliminary comparisons of continuous variables between groups. To evaluate the associations between the ureteral wall thickness (UWT) and patient- or stone-related parameters, a multivariable linear regression model was applied including both continuous predictors (e.g., age, BMI, stone volume, and stone dimensions) and binary predictors (e.g., gender, comorbidity status, stone history, and surgical history) coded as dummy variables. The model assumptions were tested: residual normality was assessed using the Shapiro–Wilk test, histogram, and Q-Q plots. Homoscedasticity was assessed using scatterplots of standardized residuals versus standardized predicted values, which showed no major pattern indicating heteroscedasticity (Figure 1). A *p*-value < 0.05 was considered statistically significant. Power analysis was conducted using G*Power 3.1 software. Based on an assumed effect size of 0.3, α = 0.05, and power = 0.80, the minimum required sample size was calculated as 88 patients. The study cohort consisted of 115 patients who underwent ureteroscopic management for impacted proximal ureteral stones at our institution.

In the regression analyses, the β coefficients represent the estimated change in UWT (mm) per one-unit increase in the predictor variable (e.g., per 1 mm^3^ stone volume, per 1 mm stone diameter, per 1 HU stone density, etc.).

The inclusion criteria were age ≥ 18 years, presence of impacted proximal ureteral stone confirmed on imaging, and availability of complete preoperative data. The exclusion criteria included age < 18 years, pregnancy, and presence of congenital or acquired urinary system anomalies. Key demographic and clinical characteristics of the cohort are summarized in Table 1.

The study protocol was approved by the Ethics Committee of Sancaktepe Şehit Prof. Dr. İlhan Varank Training and Research Hospital (Date: 14 June 2023; File No: 96).

## 3. Results

The findings obtained in 115 adult cases (37 female, 78 male patients M/F: 2, 11) undergoing ureteroscopic stone management for impacted upper ureteral stones (5–20 mm) are presented here. The mean overall patient age in the whole group was 45.35 ± 13.84 years (19–78) with a mean BMI value of 27.42 ± 3.95 (17–41.9). A total of 34 cases in our group were found to have comorbidities (31.2%), including hypertension (18%) and diabetes mellitus (21%).

The evaluation of our results revealed the following findings.

The extent and level of hydronephrosis were found to be closely correlated with the value of the UWT. As the degree of hydronephrosis increased, the mean UWT values also tended to increase in a significant manner (*p*: 0.002). On the other hand, patients with associated comorbidities also demonstrated significantly higher mean UWT values compared to those without comorbidities (*p*: 0.005) (Table 2). The positional status of the stones (horizontal or longitudinal) was evaluated, and we did not determine a significant difference between these parameters and the mean UWT values (*p*: 0.100).

**Figure 2:**
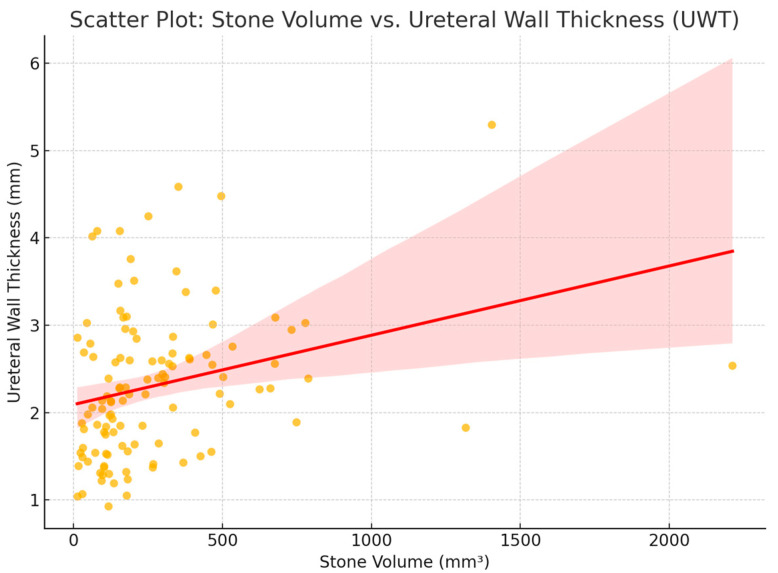
Scatterplot showing the relationship between the stone volume (mm^3^) and ureteral wall thickness (UWT, mm). A linear regression line is included to illustrate the significant positive correlation (*p* = 0.005).

Following these evaluations, a further regression analysis of the stone-related factors was performed, and this evaluation clearly demonstrated that when the stone volume and stone transverse and longitudinal diameters increase, the mean UWT values tend to increase significantly (*p* = 0.005, 0.014, and 0.005, respectively). These findings are illustrated in Figure 2, which demonstrates a positive and statistically significant correlation between stone volume and UWT (*p* = 0.005). However, there was no statistically significant correlation regarding the stone hardness or the duration of its presence (*p* = 0.614 and *p* = 0.987, respectively) (Table 3).

## 4. Discussion

Optimal management of relatively large and impacted proximal ureteral calculi constitutes a real challenge for urologists due to certain stone- and patient-related factors. In addition to other well-known stone-related parameters (size, location, and hardness), as a critical issue, the embedment of these stones in the ureteral mucosa may induce an inflammatory response in the ureteral wall, which results in increased wall thickness [10]. As a reliable and non-interventional form of evaluation, assessment of the UWT enables us to predict the extent and degree of stone impaction. The review of the existing literature indicates that the impaction status of the stone(s), in terms of increased ureteral wall thickness, may significantly influence the selection of the most appropriate treatment modality. Furthermore, its judicious clinical utility becomes more apparent when correlated with the course and outcomes of standard treatment options. Several studies have reported that increased UWT may significantly reduce the success rate of extracorporeal shock wave lithotripsy (SWL) due to impaired energy transmission through edematous and thickened tissue [4,6]. Similarly, higher UWT values have been associated with more difficult and prolonged ureteroscopic procedures, often resulting in incomplete stone removal or the need for staged interventions [9,11]. In this regard, our findings highlighting the predictive value of patient- and stone-related parameters on UWT may serve as a valuable guide for selecting the most appropriate treatment modality and anticipating procedural difficulties. This parameter may help us to predict the possible outcomes of the interventions and potential complications [9,10,11]. Furthermore, identification of such factors may allow us to monitor the course of the watchful waiting phase for ureteral calculi before the evident changes in the ureteric wall occur as the stone grows and embeds further into the ureteral wall. However, despite its crucial role, highly limited data have been reported regarding the possible effects of ureteric calculi- and body-related factors on the UWT value, which can reliably reflect the extent and severity of stone impaction.

In light of the limited data available in the literature, we aimed in this study to comprehensively assess the impact of both patient-related factors (such as comorbidities and hydronephrosis) and stone-related parameters (including volume and dimensions) on ureteral wall thickness (UWT), which serves as a surrogate marker of stone impaction. While previous studies have focused on selected predictors individually [7,8,9,10,11], our approach provides a broader, integrated analysis of multiple contributing factors in a single multivariable model.

Our results revealed that among the patient-related factors, the degree of hydronephrosis was found to be significantly correlated with the mean value of the UWT. Our results seem to be consistent with the findings of previous studies demonstrating significantly increased mean UWT values paralleling the increased degree of hydronephrosis [4]. Additionally, the presence of comorbidities, such as diabetes or hypertension, was found to be significantly associated with mean elevated values of the UWT. The inflammatory process mainly associated with these comorbidities may cause such changes in the ureteral wall thickness, possibly increasing local inflammatory changes induced by the stones [12]. The absence of a significant correlation between the mean UWT values and other patient-related factors, apart from the above-mentioned comorbidities, further supports the hypothesis that inflammation could be a major factor playing a critical role in changes in UWT [9].

On the other hand, regarding the stone-related parameters evaluated in our trial, we observed that an increase in the volume and transverse and longitudinal diameters of the stone was also associated with a positive correlation with the changes noted in the UWT. These findings seem to be in accordance with the outcomes of previous trials, which strongly suggest that the size of stones in this position could be another important contributor to ureteral wall thickening [10]. This supports the notion that a larger stone size may affect the stone’s embedment in the ureteral wall, as a result of the evident stone-induced inflammatory reaction. However, no statistically significant relationship could be demonstrated in our trial between stone hardness (measured in Hounsfield units) and UWT values, although this parameter was found to affect the impaction status in some studies [13,14]; similarly, no significant correlation was observed between stone volume and UWT (Figure 2). We believe that this relationship needs to be clarified with future studies conducted in a larger series of cases.

This inconsistency with prior studies may be attributed to several factors. First, the retrospective nature of our study may have introduced variability in HU measurements due to non-standardized imaging parameters. Second, we used manual measurement of HU at a single point rather than an averaged volumetric approach, which might limit precision. Third, other influencing variables such as the stone size, duration of symptoms, and hydronephrosis could have confounded this relationship. Finally, the sample distribution and potential heterogeneity in stone composition may also explain the lack of correlation. Further prospective studies with standardized HU evaluation are warranted to clarify this discrepancy [13,14].

Although there is some evidence in the literature suggesting a relationship between the stone location in the same part of the involved ureter and the increase in UWT [15], our findings demonstrated no meaningful correlation between the duration of symptoms and changes in UWT values. This discrepancy led us to consider that the increase in the UWT value may not originate solely from the stone presence and inflammatory response induced, but some other unidentified pathophysiological mechanisms may also influence this phenomenon. Further research is needed to explore these potential mechanisms and their contributions to the changes in UWT values, which will in turn reflect the degree of stone impaction.

Our current study may have certain limitations. First of all, the data were collected in a retrospective manner from the departmental patient records, which inherently introduces potential biases and limitations in data accuracy as well as reliability. Secondly, our sample size is relatively small, and we certainly believe that a larger series of cases would be required to confirm these results and support our conclusions further. However, in light of the highly limited data reported so far on this aspect, the findings of our current trial will contribute to recognizing and considering the parameters affecting the formation as well as the severity of the increase in the ureteric wall thickness. Another limitation of our study is that the duration of symptoms was based on patient-reported onset (e.g., flank pain). This approach is subject to recall bias, which may partly explain the lack of a significant association between symptom duration and UWT. Certain factors, such as infection status, pre-NCCT stent placement, and patient hydration status, may also influence ureteral wall thickness measurements. As these parameters were not systematically assessed in our retrospective design, they represent potential confounders. Future prospective studies incorporating these variables are warranted to clarify their possible effects on UWT. Although all UWT measurements were performed by a single experienced radiologist, which ensured standardization, inter- and intra-observer reliability were not assessed. This represents a limitation of our study, as it may influence the reproducibility of the measurements.

Based on the established role of the UWT value as a reliable predictor of stone impaction in cases with relatively larger upper ureteric calculi, as shown in our trial, the above-mentioned two factors may serve as the most clinically relevant predictors to guide treatment planning in patients with proximal ureteral stones [13,14]. This will certainly allow us to monitor the cases in a safe manner and create a proper management plan for successful interventions without complications. Stones associated with the above-mentioned contributing parameters may require early removal during the watchful waiting period to avoid further impaction, which may increase the risk of obstruction and interfere with successful procedural outcomes. Additionally, preoperative measurement of the UWT values may help to predict procedural challenges and postoperative outcomes not only for ESWL [16] but also for URS procedures [17]. Finally, integration of UWT assessment with other radiological features, such as periureteral stranding and proximal ureteral dilation, may be helpful in the development of predictive nomograms for stone impaction. These models may help identify high-risk patients in whom early intervention should be prioritized to avoid complications such as severe inflammation, fibrosis, or post-interventional stricture [18]. The findings of our current study support this concept by identifying patient- and stone-related factors contributing to increased UWT and, therefore, may help in stratifying patients in terms of procedural complexity and risk for individualized treatment planning [17,18,19,20].

## 5. Conclusions

In conclusion, our study demonstrated that greater stone volume and dimensions (length and transverse diameter) as well as the presence of comorbidities, specifically hypertension and diabetes mellitus, were independently associated with increased ureteral wall thickness in cases of impacted proximal ureteral stones. These findings highlight the importance of considering both stone characteristics and patient comorbidities in preoperative evaluation to better assess the degree of impaction.

## Figures and Tables

**Table 1:** Cohort characteristics of the study population.

Variable	Value
N	115
Age (years), mean ± SD	45.3 ± 13.8
BMI (kg/m^2^), mean ± SD	27.2 ± 3.9
Male/Female	78/37
Stone length (mm), mean ± SD	9.5 ± 3.8
Stone transverse diameter (mm), mean ± SD	5.5 ± 1.9
Stone volume (mm^3^), mean ± SD	7.7 ± 2.8
Stone density (HU), mean ± SD	969.9 ± 344.5
Hydronephrosis grades	Grade 0:6 (5.2%)
	Grade 1:21 (18.3%)
	Grade 2:50 (43.5%)
	Grade 3:35 (30.4%)
	Grade 4:3 (2.6%)

**Table 2:** Patient characteristics and UWT linear regression analysis.

Parameters	Regression Coefficient (β)	95% Confidence Interval		*p* Value
		Lower	Upper	
Gender	0.601	0.361	1.002	0.077
Age (years)	2.068	0.955	3.978	0.532
BMI (kg/m^2^)	0.267	0.633	1.534	0.166
Hydronephrosis grade	0.309	0.117	0.502	0.002
Comorbidity	2.160	1.261	3.700	0.005
Stone history	0.921	0.578	1.469	0.277
Operation history	1.123	0.690	1.826	0.401
Parenchymal thickness (mm)	0.952	0.164	1.280	0.771

**Table 3:** Stone characteristics and UWT linear regression analysis.

Parameters	Regression Coefficient (β)	95% Confidence Interval		*p* Value
		Lower	Upper	
Stone volume (mm^3^)	0.001	0.001	0.002	0.005
Stone transverse diameter (mm)	0.108	0.023	0.194	0.014
Stone length (mm)	0.068	0.021	0.115	0.005
Stone density (Hounsfield Unit)	0.001	0.001	0.001	0.614
Duration of stone (days)	0.194	0.002	0.198	0.987

## Data Availability

The original contributions presented in this study are included in the article. Further inquiries can be directed to the corresponding author.
